# Towards P-Type Conduction in Hexagonal Boron Nitride: Doping Study and Electrical Measurements Analysis of hBN/AlGaN Heterojunctions

**DOI:** 10.3390/nano11010211

**Published:** 2021-01-15

**Authors:** Adama Mballo, Ashutosh Srivastava, Suresh Sundaram, Phuong Vuong, Soufiane Karrakchou, Yacine Halfaya, Simon Gautier, Paul L. Voss, Ali Ahaitouf, Jean Paul Salvestrini, Abdallah Ougazzaden

**Affiliations:** 1 CNRS, UMI 2958, GT–CNRS, 2 rue Marconi, 57070 Metz, France; amballo@georgiatech-metz.fr (A.M.); asrvast@georgiatech-metz.fr (A.S.); ssundara@georgiatech-metz.fr (S.S.); pvuong@georgiatech-metz.fr (P.V.); soufiane.karrakchou@gatech.edu (S.K.); paul.voss@ece.gatech.edu (P.L.V.); or ali.ahaitouf@usmba.ac.ma (A.A.); jean-paul.salvestrini@georgiatech-metz.fr (J.P.S.); 2 School of Electrical and Computer Engineering, Georgia Institute of Technology, GT-Lorraine, 57070 Metz, France; 3 Georgia Tech-Lorraine, 2 rue Marconi, 57070 Metz, France; 4 Institut Lafayette, 2 rue Marconi, 57070 Metz, France; yacine.halfaya@institutlafayette.eu (Y.H.); simon.gautier@institutlafayette.eu (S.G.)

**Keywords:** h-BN, magnesium, doping, wide bandgap, heterojunction

## Abstract

Reliable p-doped hexagonal boron nitride (h-BN) could enable wide bandgap optoelectronic devices such as deep ultra-violet light emitting diodes (UV LEDs), solar blind photodiodes and neutron detectors. We report the study of Mg in h-BN layers as well as Mg h-BN/AlGaN heterostructures. Mg incorporation in h-BN was studied under different biscyclopentadienyl-magnesium (Cp2Mg) molar flow rates. 2θ-ω x-ray diffraction scans clearly evidence a single peak, corresponding to the (002) reflection plane of h-BN with a full-width half maximum increasing with Mg incorporation in h-BN. For a large range of Cp2Mg molar flow rates, the surface of Mg doped h-BN layers exhibited characteristic pleats, confirming that Mg doped h-BN remains layered. Secondary ion mass spectrometry analysis showed Mg incorporation, up to 4 × 10^18^ /cm^3^ in h-BN. Electrical conductivity of Mg h-BN increased with increased Mg-doping. Heterostructures of Mg h-BN grown on n-type Al rich AlGaN (58% Al content) were made with the intent of forming a p-n heterojunction. The I-V characteristics revealed rectifying behavior for temperatures from 123 to 423 K. Under ultraviolet illumination, photocurrent was generated, as is typical for p-n diodes. C-V measurements evidence a built-in potential of 3.89 V. These encouraging results can indicate p-type behavior, opening a pathway for a new class of wide bandgap p-type layers.

## 1. Introduction

Control of doping and conductivity is of critical importance for fabrication of semiconductor-based devices. For example, in III-nitride-based deep ultraviolet (DUV) LEDs, the p-type doping of Al rich AlGaN with Mg is extremely challenging because of its large ionization energy in AlN (~500 meV) [1,2,3,4,5]. An alternate wide bandgap material with appropriate p-type doping is highly desired. Hexagonal boron nitride (h-BN) is a promising material for p-type wide bandgap nitride such as UV-LEDs [6,7,8,9] and UV detectors [10,11,12].

Previously, it was reported that the p doping of h-BN can be achieved using Zn, Be and Mg as dopants [13,14,15,16,17,18,19,20]. More recently, contradicting experimental and theoretical studies of p-type conductivity of h-BN have been published. Experimental works report good control of p-type conductivity of h-BN by means of in situ doping of magnesium, with activation energy of 30–300 meV [7,18,19]. On the other hand, density functional theory (DFT) studies predict that p-type h-BN using Mg is most likely impossible because of the formation of small hole polarons, which increases the Mg ionization energy to more than 1 eV [21]. Other experimental studies point to defect states with energies near the valence band as a mechanism that can lead to effective doping [15]. In this paper, we report an experimental study of the electrical behavior of Mg incorporation in h-BN. In order to evaluate the potential of h-BN with Mg doping for device applications, we fabricate Mg doped h-BN/n-AlGaN heterostructures and report I–V characteristics as a function of temperature, under UV illumination and C–V measurements at room temperature.

## 2. Materials and Methods

Materials were grown in a MOVPE close-coupled showerhead 3 × 2” system on (0001) sapphire substrates using triethylboron (TEB), trimethylgallium (TMGa), trimethylaluminum (TMAl) and ammonia (NH_3_) as B, Ga, Al and N precursors, respectively. The h-BN layers were grown at 1280 °C in hydrogen ambient at 85 mbar. The growth of h-BN layers was initiated with a TEB preflow; details of these growth conditions have been reported elsewhere [22]. Biscyclopentadienyl-magnesium (Cp2Mg) was used as the source of Mg dopants. First, we grew 50 nm thick undoped h-BN, referred to as sample A, for reference. Then, we studied the effect of Mg doping on the structural and morphological characteristics of three different sets of h-BN samples referred to as B, C and D, grown at Cp2Mg molar flow rates of 0.9, 1.8 and 2.9 µmol/min, respectively. The h-BN layer thickness of the full set of samples was 50 nm, and all the other parameters including TEB molar flow were kept constant. In addition to these layers, two heterostructures made of 50 nm undoped and Mg doped h-BN grown on 500 nm thick n-AlGaN (58% Al composition, Si doped at 1.03 × 10^17^ cm^−3^) on an AlN/sapphire template were grown. The first was grown with undoped BN on n-AlGaN to be an i-n junction (sample E), and the second was grown with doped BN with the purpose of forming a p-n heterojunction (sample F). The n-AlGaN layer with 58% Al content was chosen because n-doped BN is experimentally infeasible and because it is difficult to have high quality n-doped AlN. The Cp2Mg molar flow used for sample F was the same as the one used for sample D (2.9 µmol/min). For the fabrication of samples E and F, a standard photolithography-based process was employed. First, mesa-etching isolation was achieved by inductively coupled plasma with BCl_3_/Cl_2_/Ar chemistry. Ti/Al/Ni/Au and Ni stacks were used for the n-contact and the p-contact, respectively. Each metal layer was deposited by thermal evaporation. N-contact annealing was carried out at 1050 °C for 6 s under N_2_, then the p-contact was annealed at 1020 °C for 60s under N_2_ atmosphere.

The morphological and structural properties were characterized by a scanning electron microscope (SEM) and high-resolution X-ray diffraction (HR-XRD), respectively. The HR-XRD scans were performed by a Panalytical X’pert Pro MRD system with Cu Kα radiation in triple axis mode. The FWHM value was determined for all the samples by fitting the (002) peak of the h-BN.

Secondary ion mass spectrometry (SIMS) measurements were carried out by an external company (Probion Analysis). Calibration was first performed using h-BN layers with different and controlled Mg implantation. Using this calibration data, grown samples with in situ doping were then characterized.

I–V and capacitance measurements were performed using a Keithly 4200A SMU and a Keysight E4990A impedance meter, respectively. I-V measurements were performed to verify the ohmic behavior of the contacts on h-BN samples A-D and to record the p-BN/n-AlGaN diode characteristics. Photocurrent generation measurements used a quadrupled Nd:YAG laser with a power of 10 mW at a wavelength of 266 nm as an optical source. Van der Pauw (VdP) and Hall Effect measurements were performed to determine the resistivity and doping of the grown samples.

## 3. Results and Discussions

### 3.1. Morphology and Structural Characterizations of Undoped and Mg Doped h-BN Layers

**Figure 1:**
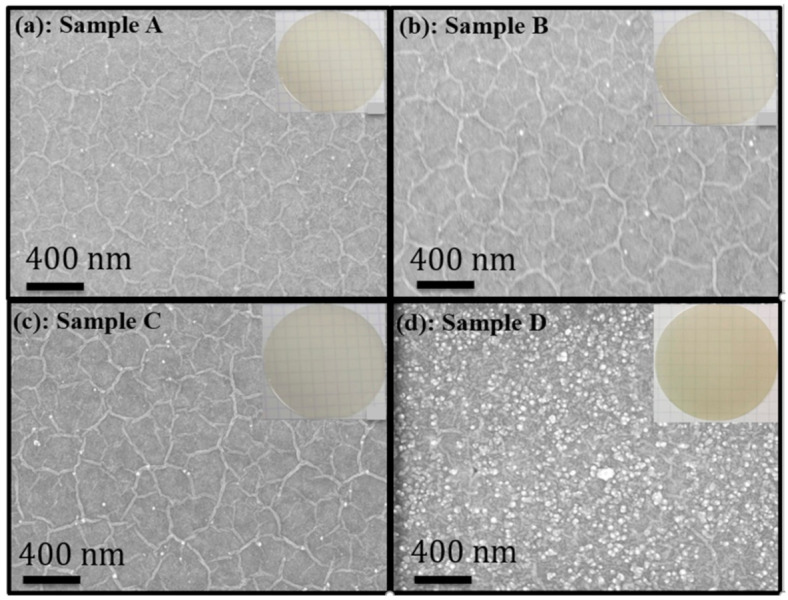
(**a**–**d**) SEM images of h-BN samples A, B, C and D, respectively. Insets show photographs of the respective samples.

The surface morphology of the undoped h-BN (sample A) and the Mg-doped h-BN samples (samples B–D) with three different Cp2Mg molar flows are shown in Figure 1. All the samples were optically transparent with a smooth surface as shown in the insets of Figure 1. Under SEM, the undoped h-BN sample exhibited an organized semihexagonal wrinkled surface (Figure 1a), which is comparable to our previous studies. Refer to [22,23] for detailed characterization. Wrinkle formation is the result of the compressive stress during sample cooling. Similar wrinkles were observed on the B and C h-BN samples (Figure 1b,c). These surface morphologies confirm that the h-BN in samples A–C is layered. On the other hand, the h-BN sample D presented in Figure 1d shows wrinkled surface morphology decorated with a high density of particles. This morphological change can be directly correlated to the heavy Mg doping. Previous studies have shown that insufficient NH_3_ flow or a parasitic reaction between the precursors may lead to grain formation, which is BN in the disordered phase (turbostratic/amorphous) [24,25,26,27]. At high Cp2Mg flow, Mg may block nitrogen atoms, leading to a low number of active N atoms in the growth front, resulting in stacking fault-induced variations in strain, which then generate a higher density of misoriented BN grains in sample D.

**Figure 2:**
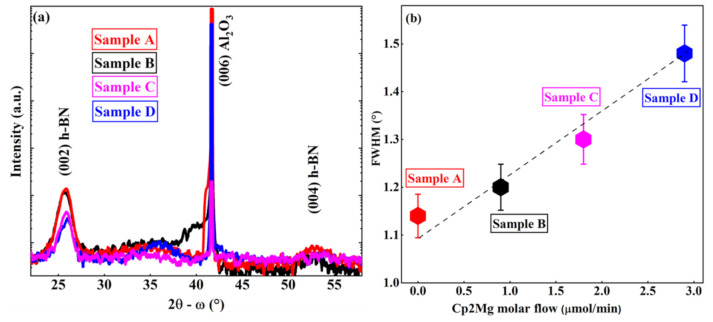
(**a**) HR-XRD 2θ-ω scans of the grown h-BN samples; (**b**) FWHM variation of (002) diffraction peak of the different h-BN samples versus Cp2Mg molar flow.

The crystalline characteristics of the as-grown samples were studied with HR-XRD 2θ-ω scan measurements as shown in Figure 2. All the samples exhibited a peak around 26° (Figure 2a) which is clearly the (002) plane reflection from the sp^2^-bonded h-BN. The (004) plane diffraction peak of h-BN was also observed at 53.5°. However, the increase in Mg led to lower intensity and broader peaks. Furthermore, when compared to the peak position of sample A (25.86°), a shift of the (002) peak was observed in sample B (25.72°). This may be related to the deformation of the surrounding lattice because of the difference of atom sizes between B and Mg [20]. However, the peak shift at the lower angle was not observed in samples C and D. This is related to the formation of an accumulation of disordered phases in samples C and D, which overcomes the effect of Mg incorporation. It is assumed that all the Mg atoms are probably not in the substitutional sites of boron. Therefore, an increase in non-substitutional Mg incorporation with the increase in Mg flow may generate additional heterogeneous strain that contributes to the increase in the FWHM of (002) reflection (Figure 2b) [28]. This increase in FWHM suggests a degradation of the crystalline quality for samples B–D. Moreover, the asymmetric shape of the (002) peak in D is likely a result of localized variation of strain in Mg doped h-BN layers, which causes formation of a large number of disordered BN particles.

### 3.2. SIMS Measurement of Mg Doping Concentration

**Figure 3:**
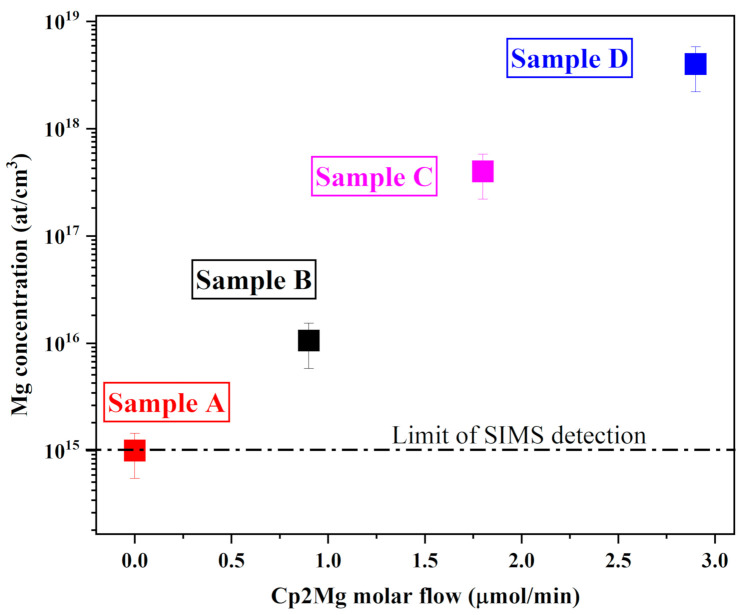
Cp2Mg molar flow dependence of the Mg concentration.

To estimate the Mg concentration in our h-BN films, secondary ion mass spectrometry (SIMS) measurements were performed. The Mg concentration increased super-linearly with the increase in Cp2Mg molar flows, as shown in Figure 3. Similar trends have been obtained in other III-V materials [29,30]. Mg incorporation reached 4 × 10^18^ atoms/cm^3^ for a Cp2Mg molar flow of 2.9 µmol/min (sample D). For the undoped sample, the value of Mg at 1 × 10^15^ atoms/cm^3^ corresponds to the limit of SIMS detection and does not correspond to a real concentration.

### 3.3. Ohmic Contact in h-BN Layers

**Figure 4:**
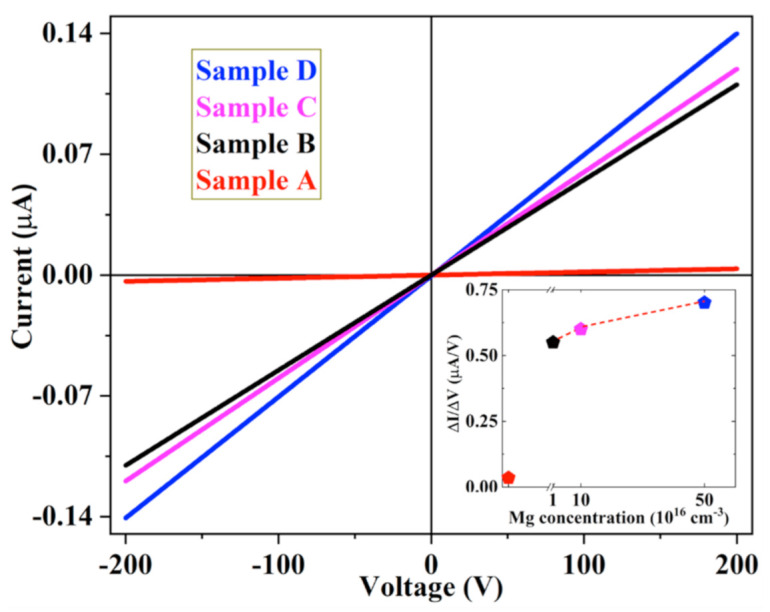
Current versus voltage measurements recorded in the undoped and Mg-doped h-BN A-D samples. The inset shows the variation of the slope of the different curves with the Mg concentration.

I–V measurements were performed on the four A–D samples. These measurements obtained information on lateral transport (perpendicular to the *c*-axis) in h-BN: the strong anisotropy of h-BN may result in different resistivity and transport in the vertical direction (parallel to the *c*-axis). Figure 4 shows the current measurement versus voltage recorded in the four h-BN samples. Whatever the sample, the I–V dependence was linear, confirming that the contacts are ohmic. It is also clear that increasing the Mg concentration results in an increase in the current for a given voltage. The dependence of the I–V slopes on the Mg concentration is reported in the inset of Figure 4. There was a large increase in the conductivity from sample A to sample B and then a slight increase from sample B to sample D. This behavior is probably related to the generation of point defects [31] with large Mg concentration in the layer, leading to a limitation (compensation) of the carrier diffusion length, especially for sample D. The resistivity of the undoped and the highly doped samples was determined from Van der Pauw (VdP) measurements performed at different temperatures (see Supplementary Materials). The resistivity was shown to vary between 1.5 × 10^6^ Ω·cm at 78 K and 2 × 10^3^ Ω·cm at 430 K for highly doped h-BN, and between 2 × 10^6^ Ω·cm at 78 K and 1 × 10^5^ Ω·cm at 430 K for undoped h-BN. At room temperature, the resistivity of the undoped sample (A) was 2 × 10^5^ Ω·cm. Compared to literature reported results, this value is much smaller. Indeed, values of 7.1 × 10^14^ Ω·cm have been reported for 50 µm thick undoped BN layer [32] and between 4 × 10^13^ and 2.1 × 10^15^ Ω·cm for h-BN ceramics obtained by a field assisted sintering technique of h-BN powders [33]. This large difference can be attributed to the degree of disorder in sintering, the difference in layer thickness but also to some residual impurities such as carbon and oxygen, as revealed by SIMS measurements (not shown here).

At room temperature, the resistivity of the sample with the highest doping (D) was 2 × 10^4^ Ω·cm, with a doping concentration of 5 × 10^15^ cm^−3^. These values can be compared to those obtained by Sun et al. [20] who reported a resistivity and doping concentration of 1.2 × 10^−5^ Ω·cm and of 1.7 × 10^14^ cm^−2^, respectively, for an h-BN monolayer grown on Cu foil and transferred on SiO_2_. For a thicker layer of h-BN (300 nm), the reported resistivity and doping concentration are 2.3 Ω·cm and 1.1 × 10^18^ cm^−3^ [7,8], respectively.

For the doped layer, an activation energy of 160 meV was derived from the slope of the Arrhenius plot (see Supplementary Materials) and attributed to the Mg level in h-BN, which is in the range of the reported values (30–300 meV) in the literature for Mg doped BN films [18,19].

The p-type conductivity was confirmed by the positive Hall voltage V_H_ derived from Hall measurement. Using this V_H_ value and the sheet resistance deduced from the VdP measurements, we determined in the highly doped sample D a p-type carrier concentration of 5 × 10^15^ cm^−3^, which is two orders of magnitude lower than the Mg atomic concentration measured by SIMS. One can then estimate the electrical activation of Mg around 1%.

In order to further investigate the p-type conductivity, we designed and studied a p-n h-BN/n-AlGaN heterojunction as discussed below.

### 3.4. Mg Doped h-BN/n-AlGaN Heterostructures

**Figure 5:**
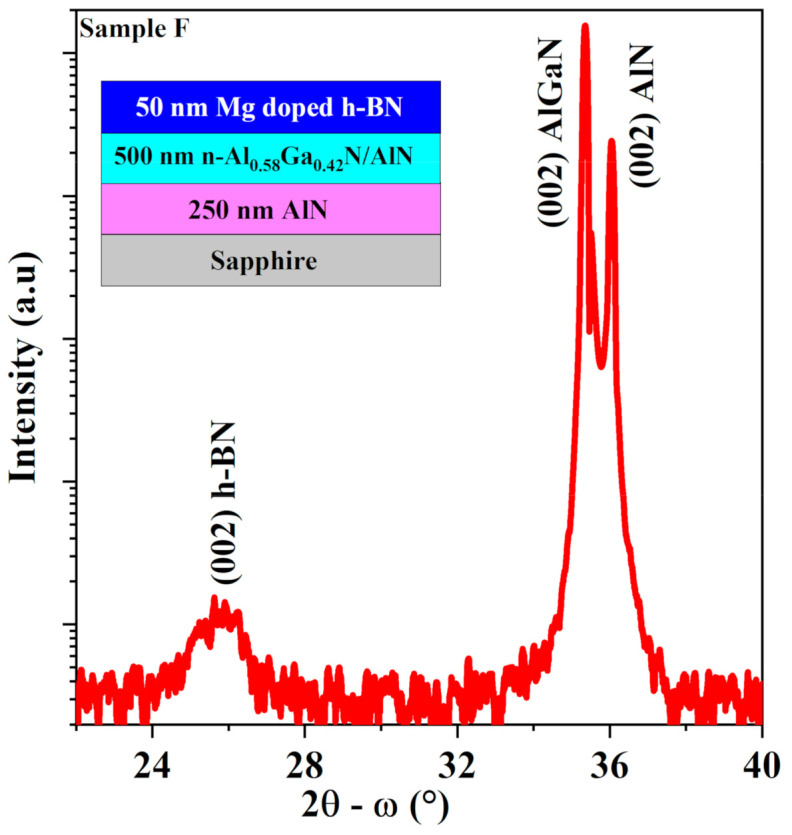
HR-XRD 2θ-ω symmetric scans of the Mg doped h-BN/n-Al_0.58_Ga_0.42_ N/AlN/sapphire heterostructures (Sample F). Inset shows the grown structure.

H-BN/AlGaN heterostructures were realized by growing undoped and Mg doped h-BN layer on Si doped Al_0.58_Ga_0.42_N/AlN/sapphire template wafer denoted by samples E and F, respectively. Figure 5 shows the measured HR-XRD 2θ-ω symmetric scan of (002) peak of the grown heterostructures. Clear diffraction peaks were identified at 25.8°, 35.3° and 36.1° which correspond to (002) reflection of h-BN, AlGaN (58% Al) and AlN, respectively. No other diffraction peak related to BN was observed by HRXRD scans indicating that the h-BN layer on AlGaN/AlN template is single phase.

**Figure 6:**
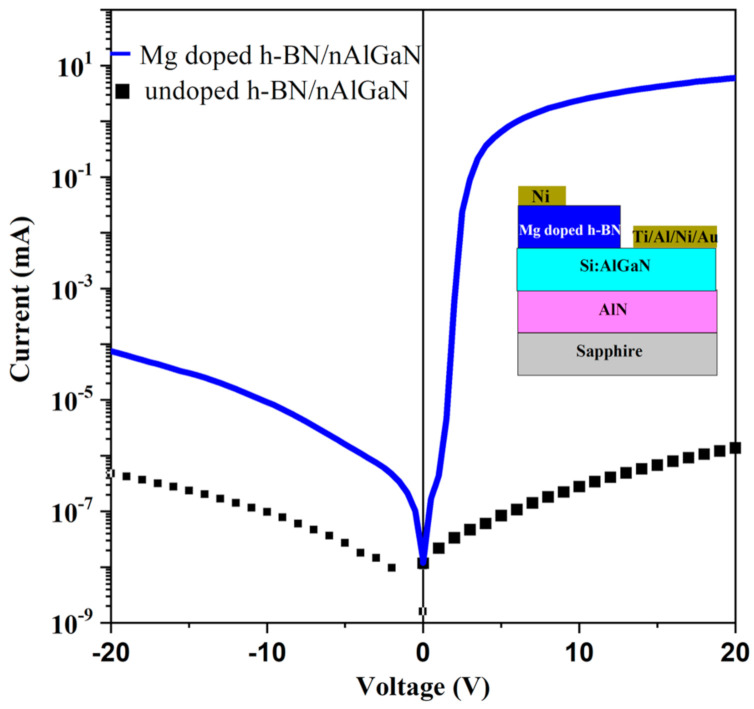
Current–voltage characteristics of undoped and Mg doped BN/n-Al_0.58_Ga_0.42_N (Samples E and F) devices measured at room temperature.

Typical I–V curves of the undoped and Mg doped h-BN on n-AlGaN heterostructures recorded at room temperature are illustrated in Figure 6. Remarkably, at forward bias, the current of Mg doped h-BN/n-AlGaN device was 7 orders of magnitude higher than that of the undoped h-BN/n-AlGaN one. In the Mg doped h-BN/n-AlGaN device (sample F), the leakage current under reverse bias voltage was reasonably low with a value of 1 µA at −10 V, but it was not saturated as expected for an ideal p-n diode, and it continued to increase with the reverse voltage. This suggests that there are leakage current paths related to the deep defect levels in the depletion layer of h-BN [34].

**Figure 7:**
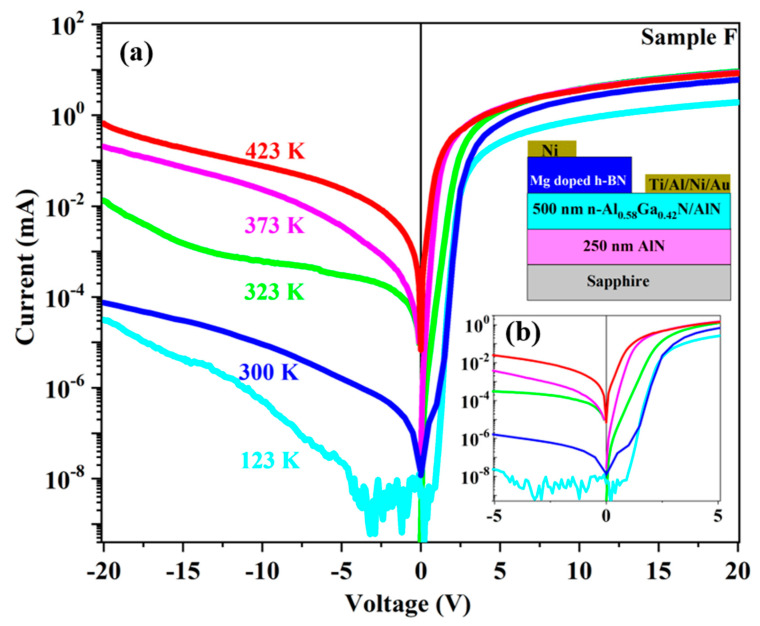
(**a**) I–V characteristics of Mg doped BN/n-Al_0.58_Ga_0.42_N (Sample F) device recorded at different temperatures. Inset (**b**): I–V zoom-in of sample F.

**Figure 8:**
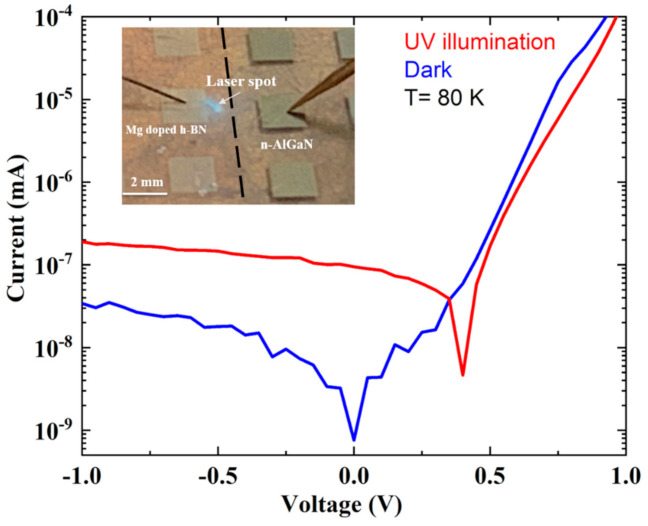
I–V curve recorded in Mg doped h-BN/n-Al_0.58_Ga_0.42_N (Sample F) at T = 80 K with and without UV laser illumination. The inset shows a photograph of the top view of the heterojunction with the electrical contact pad and the location of the laser spot.

The reverse and forward I–V characteristics of the p-hBN/n-AlGaN heterojunctions were studied at different temperature from 123 to 423 K as shown in the semilogarithmic plot of Figure 7. At forward bias, all the I–V curves exhibited strong saturation due to high series resistance, which likely originated from the resistance of the long lateral path in n-AlGaN, which had a measured carrier concentration of 1.03 × 10^17^ cm^−3^. As seen in the inset of Figure 8, the 2 × 2 mm^2^ ohmic contact pads were 3 mm away from each other, which is much larger than the 50 and 500 nm thickness of the h-BN and n-AlGaN layers, respectively, so that the resistance of the n-AlGaN layer in the lateral direction was much larger than the resistance of the heterojunction in the vertical one. As shown in Figure 7, the current increased significantly with the temperature in the range 300 to 373 K and then started to saturate at higher temperature. An inverse trend was observed for the threshold voltage. It varied from 3.2 V at 123 K to 1.5 V at 423 K. This might be due to the activation of some defects with the temperature increase. This assumption is consistent with the increase in the current at reverse bias with the temperature. For instance, at −5 V, the leakage current increased from 30 pA at 123 K to 0.2 mA at 423 K.

As the heterojunction showed better I–V characteristics at low temperature (low current leak), a set of P-N diodes were cooled to liquid nitrogen temperature and illuminated under UV laser light at 266 nm. Illumination occurred on the p-side of the junction through the Mg doped h-BN, next to the contact pad, as shown in the inset of Figure 8. As the 266 nm laser was below the bandgap of h-BN and above the bandgap of Al_0.58_Ga_0.42_N, UV absorption primarily occurred in the Al_0.58_Ga_0.42_N. Even with low output power laser of 10 mW, the partial absorption of the glass window of the cryostat, and the likely low collection efficiency hindered by the lateral transport in both h-BN and AlGaN, a significant photocurrent (120 pA at 0 bias voltage) was obtained. The generation of a photocurrent is evidence of the existence of built-in electrical field at the junction between Mg doped h-BN and n-AlGaN.

**Figure 9:**
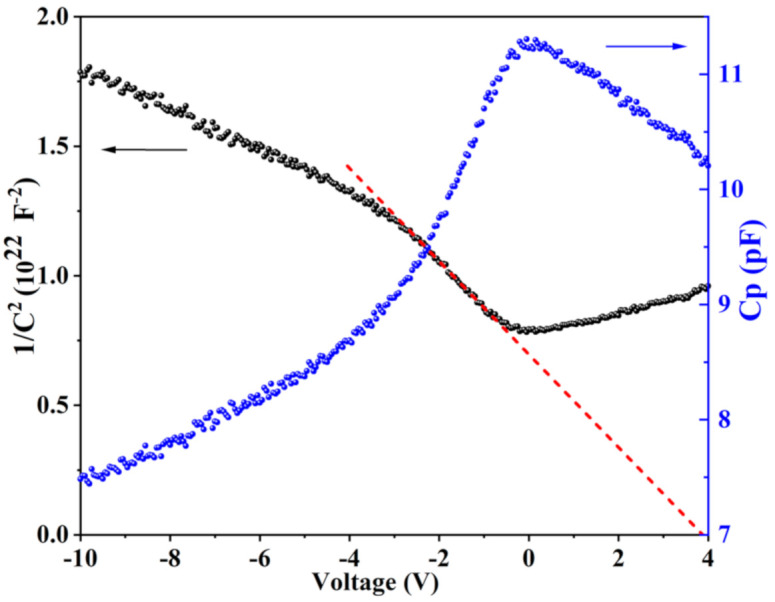
Capacitance voltage measurement in Mg doped h-BN/n-Al_0.58_Ga_0.42_N (Sample F) measured at 1 MHz and room temperature, and corresponding 1/C^2^ plot versus voltage.

CV measurements at room temperature and 1/C^2^ plot versus voltage are both shown in Figure 9. For forward voltage, the capacitance measurement was limited to small voltages since the forward bias current and the diffusion capacitance affect the accuracy of the capacitance measurement. For reverse applied voltage, the capacitance decreased, as expected for a p-n diode, since the depletion region width increased accordingly. At larger negative voltage, the capacitance did not saturate, which indicates a moderate p-doping concentration (here, we assume that the n-doping concentration of the AlGaN layer is much larger than the p-doping concentration of the h-BN layer, leading to a depletion mostly located in the h-BN layer). The dotted line in the 1/C^2^ plot formed a reasonable linear fit at voltage close to zero from which one can conclude that the doping concentration was almost constant close to the Mg doped hBN/n-AlGaN interface. Extrapolation of the low voltage 1/C^2^ measurements (see Figure 9) resulted in a built-in voltage of 3.9 V, consistent with the bandgap of the Al_0.58_Ga_0.42_N material, and a small discontinuity between the valence bands of h-BN and AlGaN as reported in XPS heterostructure experiments [35]. It is worth mentioning that the measured capacitance was much lower than that expected for a 2 × 2 mm^2^ surface area of the device. This could be attributed to either a partial oxidation of the Ni pad (the current spread in the pad has been noticed to decrease with time) leading to a decrease in the effective surface area and/or a large resistance of the AlGaN path, which attenuates the AC capacitance signal. This makes extraction of the p doping from the C-V measurements complicated.

## 4. Conclusions

In summary, we used MOVPE to grow h-BN layers with different Cp2Mg molar flow rates. The van der Waals nature of the Mg-doped h-BN layers was retained up to a certain threshold value of the Cp2Mg molar flow. The electrical conductivity of Mg-doped h-BN layers increased with Mg concentration, showing the possibility to control conductivity of h-BN. We demonstrated epitaxial growth of Mg-doped h-BN layers on Al rich n-AlGaN layers, which exhibited diode-like I–V characteristics. More importantly, the illumination of p-BN/n-AlGaN heterojunction generated significant photocurrent, which is very convincing evidence of the p-n junction formation between p-BN and n-AlGaN. C–V measurements were also consistent with a built-in voltage around 4.0 V. These results are very encouraging for further research and may open potential applications in wide bandgap optoelectronic devices such as deep UV LEDs, deep UV lasers and novel neutron detector structures.

## Data Availability

The data that support the findings of this study are available within the article or from the corresponding author upon reasonable request.

## Supplementary Materials

See Supplementary Materials for the resistivity measurement of sample D and I-V characteristics of Samples F in the attached file. The following are available online at https://www.mdpi.com/2079-4991/11/1/211/s1, Figure S1: Resistivity of the 50 nm thick undoped and Mg-doped h-BN (sample D) versus temperature. Figure S2: Activation energy of the 50 nm thick Mg-doped h-BN. Figure S3: I-V characteristics measured on two Mg doped BN contact pads and two n-AlGaN contact pads.
