# Supplementary material for: Towards P-Type Conduction in Hexagonal Boron Nitride: Doping Study and Electrical Measurements Analysis of hBN/AlGaN Heterojunctions
Source: Nanomaterials (Basel). 2021 Jan 15;11(1):211. doi: 10.3390/nano11010211 (PMC7829971; doi:10.3390/nano11010211)
Supplement: Supplementary file 1 [file nanomaterials-11-00211-s001.pdf]

Supplementary Figures:

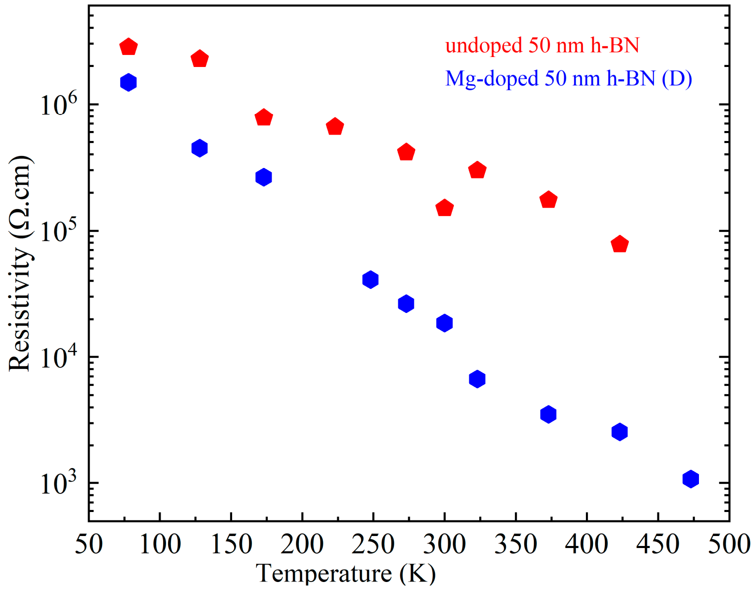

Figure S1: Resistivity of the 50 nm thick undoped and Mg-doped h-BN (sample D) versus temperature.

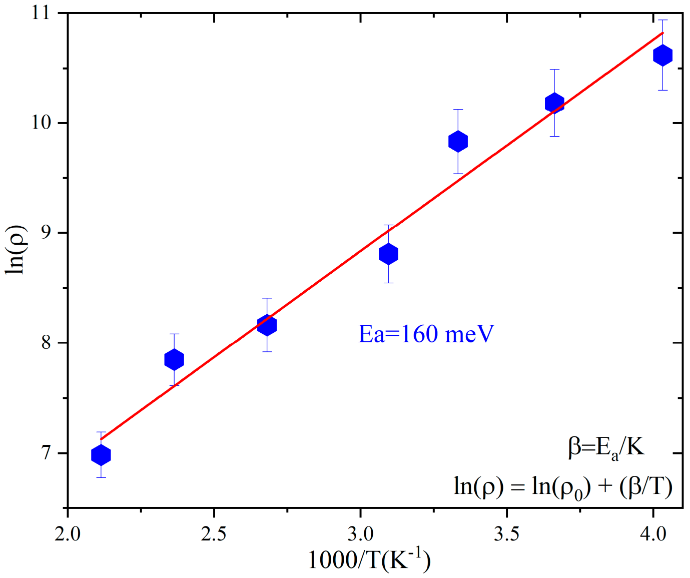

Figure S2: Activation energy of the 50 nm thick Mg-doped h-BN.

The nature of the metal contacts on each junction side has been checked. As shown on Figure S3a and Figure S3b, a linear curve is obtained in each case revealing an ohmic behavior.

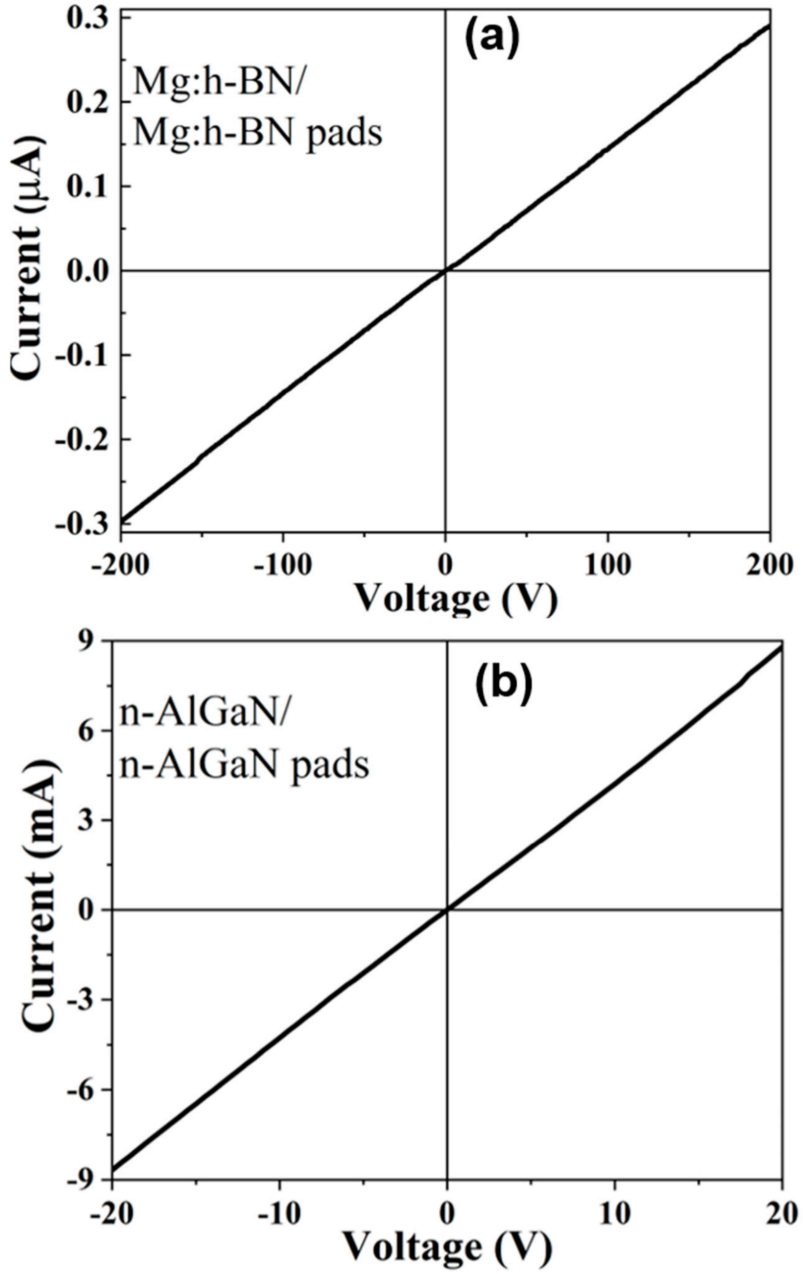

Figure S3: (a), (b): I-V characteristics measured on two Mg doped BN contact pads and two n-AlGaN contact pads.
